# Hypocrisy in ethical consumption

**DOI:** 10.3389/fpsyg.2022.880009

**Published:** 2022-08-25

**Authors:** Colin Foad, Geoff Haddock, Gregory Maio

**Affiliations:** ^1^School of Psychology and Clinical Language Sciences, University of Reading, Reading, United Kingdom; ^2^School of Psychology, Cardiff University, Cardiff, United Kingdom; ^3^School of Psychology, University of Bath, Bath, United Kingdom

**Keywords:** hypocrisy, ethical consumption, frugality, anchoring, moral decision making

## Abstract

When making consumption choices, people often fail to meet their own standards of both ethics and frugality. People also generally tend to demand more of others than they do of themselves. But little is known about how these different types of hypocrisy interact, particularly in relation to attitudes toward ethical consumption. In three experiments, we integrate research methods using anchoring and hypocrisy within the context of ethical consumption. Across three experiments, we find a default expectation that people (particularly people other than ourselves) should spend less on consumer items than they actually do. This default position can be inverted by making the ethical context of consumption salient, whereby the expectation is then that people (particularly other people) should spend more on consumer items than they actually do. Experiments 2 and 3 show that a moderate price anchor for ethical consumption is sufficient to shift expected standards for other people, but a higher price anchor is required to shift expected standards in personal behaviour. We discuss the countervailing roles of frugality and ethical consumption in understanding hypocrisy and ethical decision-making.

## Introduction

Imagine somebody is out shopping for a new pair of jeans. Their first instinct might be to be sensible and not spend too much money. They find what they think is a bargain. Later that day, they show off their new jeans to their friends. One of their friends admires the purchase, but another asks them if the low price might clash with their own personal attitude toward workers’ rights. Suddenly, the context has shifted their mindset from a focus on frugality (I should save money) to a focus on the fairness of manufacture (I should spend in line with my ethical standards), leading them to consider how they should balance frugality and ethics. In addition, they then wonder how other people juggle these views when shopping. This article empirically explores the discrepancies people hold in terms of motives to spend more or less, depending upon their desires for frugality and ethics, and how these discrepancies differ for themselves and others.

## Frugality

Despite being widespread across cultures, frugal dispositions are relatively understudied in psychological and consumer research. Historically, frugality has been operationalised in diverse ways, but it generally encompasses a broad motivation toward consuming and spending less, and people’s default position is usually to spend as little as possible when shopping (Dahl et al., 2003). Of course, in some contexts, feelings of success and status can encourage a desire for luxury goods (Mandel et al., 2006), but even in these situations, people would likely happily accept a discount if offered. Whilst concepts of frugality also often contain an aspect of maximising quality for the cost paid, our psychological operationalisation here specifically focuses on the *motivation* to spend less—avoiding that “pain” or guilt that is associated with spending money (Rick et al., 2008).

The diversity of definitions of frugality leads to a diversity of found effects. Frugality predicts less materialism (Goldsmith and Flynn, 2015), greater energy reduction (Fujii, 2006), and more concern for how items are used at the end of their life (Evers et al., 2018). However, frugality also predicts less purchasing of environmentally friendly products (Wang et al., 2021). Conspicuous consumption, an antonym to frugality, can be framed as either morally permissible or objectionable depending on the values that are salient at the time of the behaviour (Goenka and Thomas, 2020). Understanding the relationships between frugality motives and sustainable behaviours thus needs further research, particularly given the potential impact of the COVID-19 pandemic on people’s attitudes towards money, materialism, and consumption (Moldes et al., 2022). To address this issue, this research directly contrasts the desire to be frugal, in terms of spending less, and the desire to act ethically.

### Ethical consumption motives

Whilst the motivation to spend less is powerful, and the market share of most ethical producers is comparatively small, consumers will also be reluctant to buy products if the consequences of the manufacturing process conflict with their moral beliefs. Such concerns have led to growing markets in goods ranging from Fairtrade coffee to sweatshop-free jeans. The demand for these goods stems from the fact that modern marketplaces are replete with items sold through intricate production chains, and consumers often seek evidence (e.g., product certification) to help them act in line with their ethical concerns (Chatzidakis et al., 2007; Gillani et al., 2019). These potential conflicts between price and morality can lead to different consumer expectations dependent upon whether they perceive the exchange as taking place in a “moral economy,” where societal needs should be prioritised, or a “market economy” where the self-interest of the consumer is expected to be maintained (Campbell and Winterich, 2018). However, it is worth noting that the consumer relationship with ethical labels such as Fairtrade is a nuanced one, as consumers may over-simplify their understanding of such certification into a binary perspective whereby “Fairtrade equals ethical,” when the reality for the producers is much more complex (Herman, 2010).

Research has found that people are willing to pay a higher monetary price to consume ethically. For example, people are willing to receive lower investment returns (up to $5,000 over 15 years) as a result of choosing ethical funds (Lotz and Fix, 2014) and pay a 46% premium for natural (organic) wine (Galati et al., 2019). Furthermore, a majority of United States participants were willing to pay an extra 25% on a sweater if it was ethically produced (Hertel et al., 2009), and field research demonstrated that people were willing to pay a 10% premium for a Fairtrade label on coffee (Hainmueller et al., 2015). More generally, ethical spending, personal boycotts, and ethical investment have all seen rapid growth in recent years (e.g., Carrigan et al., 2004; Webb et al., 2014). However, it is worth noting that a substantial intention-behaviour gap is often found (Carrington et al., 2014), consumers can hold different moral considerations for the same products (Luttrell et al., 2021), and people sometimes choose to avoid ethical information when consuming (Reczek et al., 2018). There are thus competing motives at work when people consider the ethics of consumption.

Of course, one crucial barrier to ethical consumption is cost. People often encounter trade-offs between cost and ethical standards (Carrigan and de Pelsmacker, 2009). Price is typically indicated as the primary concern in opting for a more ethical alternative (Bray et al., 2011). However, it is not yet clear how people use available information to resolve such dilemmas because we do not use numbers proportionately to make ethical judgements. For example, when people were asked how much money they thought should be spent on saving waterfowl from environmental hazards, responses did not differ when the number of birds was 2,000, 20,000, or 200,000 (Desvousges et al., 1993). In theory, this difficulty arises partly because of the fungibility problem—people sometimes have difficulty translating moral concerns into monetary value (McGraw et al., 2003). People’s use of pricing in ethical purchasing, therefore, requires further research.

### Anchoring and prices

One potential core determinant for understanding the role of price in ethical decision-making comes from Tversky and Kahneman’s (1974) seminal work on how judgements can be influenced by numerical anchors. The anchoring mechanism is a well-established heuristic in decision-making processes, whereby people use an initially presented number to guide their subsequent judgements (Furnham and Boo, 2011). This influence includes the effects on the perceived suitability of personal-injury rewards (Marti and Wissler, 2000), expert judgements regarding appropriate criminal sentencing (Englich et al., 2006), and a range of forecasted behaviour (Critcher and Gilovich, 2008) and actual behaviour (Cheek et al., 2015). Arbitrary anchors also affect judgements of everyday items’ prices (Ariely et al., 2003; Simonson and Drolet, 2004), and marketing techniques use higher anchors to increase the quantity of purchases in a supermarket (Wansink et al., 1998). Further, anchoring effects are not simply limited to individuals unfamiliar with the content of the task judgement; experts are also susceptible. For example, car dealers reported anchor-directed estimates for the value of a used car, although these effects could be attenuated by encouraging the dealers to generate reasons why the initial anchor might be inappropriate (Mussweiler et al., 2000). Taken together, this evidence shows the important role of anchoring effects in a range of judgement and purchasing contexts.

However, in consumption situations with an ethical component, an unanswered question is whether setting high-price anchors is effective in promoting changes in expectations. There are competing possibilities regarding how anchor size for ethical consumption may influence decision-making. If people are told they need to pay a much higher price, they might be more motivated to act because they see how far away they are from their normative (ought) moral standard, or they might be demotivated because the price is seen as unattainable (e.g., Schill and Shaw, 2016). In addition, high-price anchors may convey a prescriptive norm that is unaffordable for most people, and this unattainable standard can also demotivate compliance (Cialdini, 2003; Burchell et al., 2013). Alternatively, if people are told they only have to pay a bit more, they might be motivated to act because they only need to shift their current position a comparatively small amount; however, faced with such information, they might also simply assimilate the higher anchor into their perception of their own current behaviour (Strack et al., 2016) and thus see no need to change. So, whilst the research on anchoring suggests it impacts on people’s understanding of pricing, it is important to examine the role of anchors directly in the context of ethical consumption.

### Ethical consumption and hypocrisy

Ethical consumption involves balancing motives of frugality with moral concern and can elicit many types of tension (Pecoraro and Uusitalo, 2014). Dissonance can arise from perceived differences between what we *should* do versus what we *want* to do (Milkman et al., 2008). Accordingly, the ethical context can lead people to reflect on the potential for hypocrisy. Further, such hypocrisy can exist at an *intrapersonal* or *interpersonal* level. The intrapersonal level represents discrepancies between how an individual thinks *they themselves* should and would react in a particular situation (see Monteith and Voils, 1998), whereas the interpersonal level represents discrepancies between what people demand of themselves versus what they demand of *other people* in the same situation (see Stone and Fernandez, 2008).

The existence of such intrapersonal and interpersonal discrepancies has been widely studied. For example, people often generally fail to meet their best intentions (Kruger and Gilovich, 2004), and egocentric biases are susceptible to anchoring and adjustment processes, whereby people use their own perspective to then judge what they expect of others (Epley et al., 2004). In terms of judging others’ consumption choices, people tend to use other people’s income levels to inform their judgements of how necessary and permissible they think consumer items are (Hagerty and Barasz, 2020). In the context of ethical consumption specifically, some people will actually denigrate others who consume ethically (Zane et al., 2016), and judge lower-income individuals’ ethical choices as *less* moral compared to a conventional alternative (Olson et al., 2016).

Two studies of hypocrisy are of particular relevance to our focus on ethical consumption. First, Paharia et al. (2013) found an effect of interpersonal hypocrisy—participants considering a vacation for themselves were more accepting of relevant questionable labour practices than participants considering an equivalent vacation for friends. Second, Gillani et al. (2019) found a moderating effect of intrapersonal hypocrisy in ethical consumption, whereby higher dispositional hypocrisy (e.g., I fail to practice what I preach) led to a weaker relationship between psychological proximity (e.g., I can identify with the poor workers producing Fairtrade products) and engagement with the Fairtrade movement (e.g., I often participate in activities relating to Fairtrade).

Whilst all of these studies are informative in showing the predictors of how people judge others’ consumer choices, they exclusively rely on judgements of specific purchasing choices in terms of morality, necessity, and permissibility. A core aim of our research was to examine whether potential discrepancies between expectations of the self and others could be framed in terms of financial costs, by allowing participants to judge the actual prices they think people do, and should, spend.

### Present research

Our research, therefore, builds on existing findings in three important ways. First, it identifies if people hold a default motive toward frugality when simultaneously evaluating their ought and actual (*intrapersonal hypocrisy*) consumption behaviour, operationalising this motive using price as the dependent variable. Second, it examines the willingness to demand more of others than of the self (*interpersonal hypocrisy*), both in terms of frugality and ethical concern. Third, more broadly, it demonstrates how intrapersonal and interpersonal hypocrisy can be combined in an experimental setting, using between-participants and within-participants designs, and using prices, rather than moral judgements, to more directly access people’s consumer perceptions.

For each experiment, “*should-actual*” spend differences were computed by calculating the difference between what people thought they should (or people generally should) spend on an item, and what they actually spend. The differences represent a motive to act more frugally (should spend less than actual payments) or more ethically (should spend more than actual payments). To examine how ethical information can shift people’s expectations from their default position, the should–actual differences were compared against a control condition (self or people generally). The analyses thus focus on the magnitude of difference between the control condition and each level of ethical information. The primary measures assessed should–actual differences regarding the products targetted in the ethical information, whilst the secondary measures assessed responses to other products. These latter measures were used to test for potential spillover effects to products that were close or distant in relevance to the target product.

Our three experiments tested five overarching hypotheses. First, participants who do not see any ethical information (the control conditions) will default to a motive of frugality, that is, they will state they, and others, should spend *less* than they actually do. Second, participants will require more frugality from others compared to themselves (i.e., a larger should–actual gap for *people generally*, compared to the *self*). Third, this default motive of frugality will be inverted by providing ethical information, such that participants who see ethical information will state that they, and others, should spend *more* than they actually do. Fourth, the inversions toward ethical concern will be greater for participants’ expectations of others’ behaviour than for themselves—that is, participants’ shift in expectations of frugality (should spend less) to ethical concern (should spend more) will be larger for *people generally* than for the *self*. Fifth, a high-price anchor will elicit a greater shift away from frugality than a moderate anchor.

Power calculations were not performed prior to data collection, because the effect sizes for our novel methodology were unknown. The sample sizes were instead set in line with available resources and contemporary experiments with comparable methods (e.g., Paharia et al., 2013). Twenty participants per cell were considered sufficient for Experiment 1 as we anticipated medium effect sizes for comparisons between ethical information and control conditions. The sample size was increased to 30 participants per cell for Experiments 2 and 3, as we introduced more subtle differences between price anchors across conditions.

## Experiment 1

Experiment 1 was used as an initial test of the first four of our hypotheses, to discover the extent to which the ethical information provided influenced participants’ judgements, and to inform what would be suitable in terms of price anchors for the following experiments.

### Method

#### Participants

Eighty-three students at a UK University (74 women, 9 men; *M*_age_ = 18.78, range 18–26 years) participated for course credit.

#### Design and procedure

Experiment 1 utilised a 2 (ethical information: present vs. absent control) × 2 (target: self vs. people generally) between-participants design. Participants were randomly assigned to one of the four cells. Participants completed the experiment individually on a computer in a laboratory.

Participants initially reported their gender, age, and how much they spend on clothes in an average month. Next, they either saw a video that made the ethical consequences of cheap clothing salient or (in the control condition) they proceeded directly to the dependent measures. Participants in the “self” conditions were also asked to briefly describe the last time they bought jeans and t-shirts; these open-ended responses were used to identify participants who were potentially atypical (e.g., bought jeans from a charity shop, and thus could be referring to a context where paying more effectively meant donating more to charity). The supplementary measures followed the dependent measures. Unrelated to this paper, other measures included the Schwartz Values Survey (SVS; Schwartz, 1992), the Identification with All Humanity Scale (IWAH; McFarland et al., 2012), and the Humanity Esteem Scale (Luke and Maio, 2009), which are noted for procedural completeness. Finally, participants were asked for an estimation of their financial security and an estimation of how much a typical student spends on a pair of jeans. Financial security was not found to moderate the effects reported in our experiments and is thus not included in the analyses.

#### Ethical information

Participants in the ethical information condition viewed a two-minute video presentation of text and images designed as a ‘charity infomercial’ for these experiments. Links to all the videos used in this project are available in Supplementary Materials. The video presented text asking the viewer to consider where clothes are bought and where they are made. These prompts were interspersed with relevant images (e.g., a shopping centre and a factory). The information indicated that prices in the United Kingdom are often very low and that the Environmental Justice Foundation suggests there are serious negative consequences of such low prices. The Environmental Justice Foundation is a real organisation that campaigns against child labour. Text prompts then asked the viewer to consider how much we actually pay for our clothes and how much we should pay. The video was designed to make the issue salient and engaging for the participant, without eliciting a strong affective response (e.g., images used did not show workers in distress). Discussions with participants in the debriefing sessions suggested these aims had been achieved.

#### Dependent measures

##### Should–actual differences

Participants responded to items by asking how much they (or people generally) *should* and *actually* spend on an average pair of jeans, t-shirt, and box of tea.^1^ In this experiment, our manipulation was specifically about clothing, so the two clothing items were combined into single indices of actual and should clothes purchasing in this experiment (*r*s = 0.46–0.50, *p*s < 0.001).

##### Supplementary measures

Participants were also asked how much they should and actually donate to charity, to offer an alternative comparison behaviour that may have been impacted by the manipulation. Additionally, participants read a vignette describing a student who purchased a pair of inexpensive jeans at full price for £8.50 ($13).^2^ Participants rated the moral acceptability of this purchase (1 = *morally wrong*, 11 = *morally right*). These exploratory measures were not central to the main hypotheses, and the associated findings are available in Supplementary Materials.

### Results and discussion

#### Frugality in the control conditions

**FIGURE 1:**
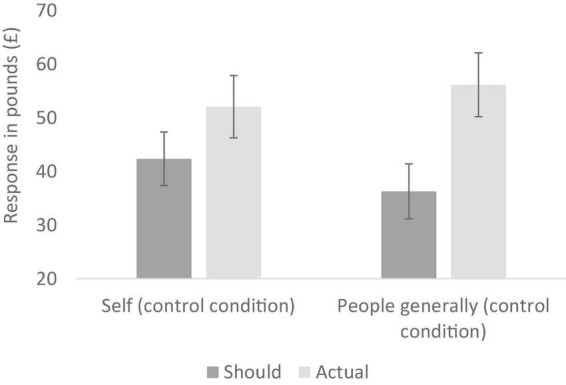
Reported should and actual spends for clothing in the two control conditions (Experiment 1); error bars show 95% confidence intervals.

In the control (no ethical information) conditions, a mixed design analysis of variance (ANOVA) was conducted with target (self vs. people generally) as the between-participants variable and should vs. actual ratings for the two clothing items combined as the repeated measures variable. This analysis revealed no main effect of target (*p* = 0.743), but a significant main effect of should vs. actual ratings, *F* (1, 39) = 39.66, *p* < 0.001 *partial*η^2^ = 0.50. This reflects the predicted default position of wishing to spend less on clothes than is actually the case (i.e., frugality). This main effect was qualified by a significant interaction, *F* (1, 39) = 4.68, *p* = 0.037, *partial*η^2^ = 0.11. As seen in Figure 1, the simple-effects analyses showed that participants thought they should spend (*M* = 42.38, *SD* = 12.04) a bit less than they actually do (*M* = 52.10, *SD* = 13.16, *p* = 0.005), but that people generally should spend (*M* = 36.30, *SD* = 10.52) a lot less than they actually do (*M* = 56.18, *SD* = 13.15, *p* < 0.001) (All simple-effects analyses in this paper use the Bonferonni correction for multiple comparisons). These data support our first two hypotheses: without ethical information being salient, (1) people default to a desire to be frugal and (2) people believe they are more frugal than others.

#### Effects of ethical information

**FIGURE 2:**
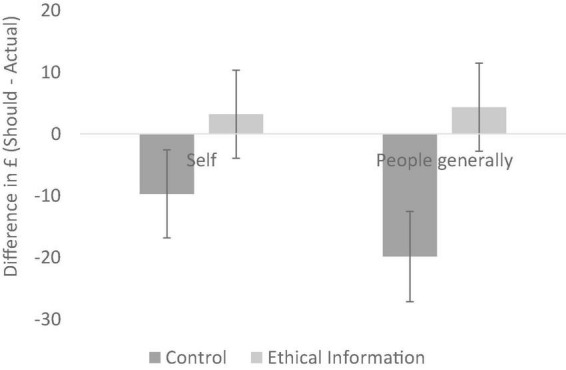
Should–actual differences for clothing (Experiment 1); error bars show 95% confidence intervals.

We conducted 2 (ethical information vs. control) × 2 (self vs. people generally) between-participants ANOVAs on should–actual differences for our primary consumer items (clothes). The ANOVA revealed a significant main effect of ethical information, *F* (1, 79) = 26.49, *p* < 0.001, *partial*η^2^ = 0.25. The main effect of target (*p* = 0.215) and the interaction was non-significant (*p* = 0.121). As seen in Figure 2, the ethical information shifted participants from a position of frugality to a position of ethical concern, for both the self (*MD* = 12.91, *p* = 0.013, *partial*η^2^ = 0.08) and for people generally (*MD* = 24.21, *p* < 0.001, *partial*η^2^ = 0.22). These data support our third hypothesis—the ethical information was effective in shifting participants concern away from frugality and toward ethical concern. However, whilst the pattern of data was as expected, insofar as the mean change was greater for people generally than the self, the non-significant interaction does not support our fourth hypothesis—that the shift from frugality to ethical concern would be larger when the target was *people generally* compared to when the target was the *self*.

The analysis of responses in the control conditions revealed a clear default position in participants’ understanding of their consumption. As predicted, there was a consistent desire to be more frugal—supporting our first hypothesis. Also, when no ethical information was provided, the results revealed evidence of interpersonal hypocrisy. *People generally* were seen to be less frugal than the participants themselves—supporting our second hypothesis. However, this frugality motive was significantly influenced by ethical information. Making salient social justice issues in the manufacturing of cheap clothing caused participants to significantly increase the amount they *should* spend, relative to how much they *actually* spend – supporting our third hypothesis. However, the expected interaction between target and ethical information was not significant; hence, our fourth hypothesis was not supported by these data.

## Experiment 2

Experiment 1 showed a default preference for frugality, the tendency to see others as more profligate than ourselves, the efficacy of ethical information in inverting these positions, and data upon which to base price anchors for our next experiments. This information was used to guide the design of Experiment 2, allowing us to test whether setting particular anchors led to different expectations for the self and other people. A price anchor for ethical consumption was set just above, or much more above, current price estimates. This enabled us to test whether moderate or higher price anchors were more effective in shifting people from a default preference for frugality to one of ethical concern. Our fifth hypothesis could thus be examined for the first time, whilst testing our four other hypotheses again.

Using the data from Experiment 1, the moderate anchor was set as £31 for a pair of jeans (a 22% increase in the perceived normal price found in Experiment 1, £24), congruent with the amount that prior research has found people are willing to pay for ethical clothing (see Hertel et al., 2009). The high anchor was set as approximately double the moderate anchor (£61) because this was considered to be a realistic scenario (i.e., around the price of premium brand jeans in the United Kingdom), but a large deviation from current norms. It is hence plausible that the high anchor will be more effective than the moderate anchor at changing perceptions of the acceptable price to pay for a product.

Whereas Experiment 1 used a between-participants design to test for differences between self and people generally, Experiments 2 and 3 used a within-participants design for this variable. There are two reasons for this design choice. First, it allows us to *directly* assess whether people are explicitly willing to report different expectations for themselves and people generally. Second, it allows us to maintain greater statistical power compared to a between-participants design in the sample we had available.

### Method

#### Participants

Ninety students at a UK University (76 women, 14 men; *M*_age_ = 19.28, range 18–22 years) participated for course credit. Three participants were excluded from the analyses because they reported recent clothes purchasing from charity shops, and such purchases do not subsume the same tensions between personal costs and ethical costs that are required for our research questions.

#### Design and procedure

A mixed design was used. The between-participants independent variable had three levels of ethical information: no information, moderate anchor, and high anchor; the within-participants independent variable was target (self vs. people generally). Participants were randomly assigned to one of the between-participant conditions. The same dependent variables and supplementary measures were taken as in Experiment 1.

Participants completed the experiment individually in a laboratory and received the same demographic items as in Experiment 1. They then either saw a video containing the moderate anchor, the high anchor, or in the control condition simply proceeded to the dependent measures. Participants completed the same ethical consumption items from Experiment 1, but this time, participants responded to the items for themselves *and* for people generally. Finally, they completed the same two final items from Experiment 1, assessing personal financial security and their perception of the price of an average jeans purchase.

#### Ethical information

Participants were presented with the same ethical information video as in Experiment 1, with additional slides containing the price anchor information. Participants in the moderate anchor condition viewed information purporting to be from a non-governmental organisation that had calculated £31 as the minimum price at which jeans could be ethically produced. Participants in the high anchor condition viewed the same slides, but the anchor was set as £61.

#### Dependent measures

Participants indicated the prices that they should and actually pay for each product, using the same scales as in Experiment 1, followed by ratings of the prices that other people should and actually pay for each product.

### Results and discussion

#### Frugality in the control condition

**FIGURE 3:**
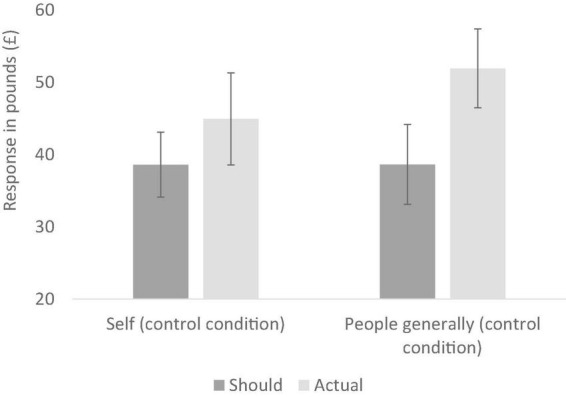
Reported should and actual spends for clothing in the two control conditions (Experiment 2); error bars show 95% confidence intervals.

In the control condition, we conducted a two-way repeated measures ANOVA with a target (self vs. people generally) and type of judgement (should vs. actual spend on clothes) as the repeated measures variables. As seen in Figure 3, participants thought they should spend (*M* = 38.59, *SD* = 11.37) a bit less than they actually do (*M* = 44.93, *SD* = 16.12) though this difference was not significant [*F* (1, 26) = 2.11, *p* = 0.158, *partial*η^2^ = 0.08]. In contrast, participants thought *people generally* should spend (*M* = 38.63, *SD* = 13.98) a lot less than they actually do (*M* = 51.93, *SD* = 13.78), and this difference was significant [*F* (1, 26) = 25.91, *p* < 0.001, *partial*η^2^ = 0.50]. However, the interaction was not significant [*F* (1, 26) = 2.88, *p* = 0.102, *partial*η*^2^* = 0.10], hence yielding limited support for our first two hypotheses.

#### Effects of ethical information

The anchors made jeans salient within the ethical information. Consequently, jeans became our primary variable of interest, with t-shirts now a spillover variable. We thus conducted a 3 (ethical information: absent, moderate anchor, high anchor) × 2 (target: self vs. people generally) mixed ANOVA on should–actual differences for each item.

##### Primary variable (jeans)

The ANOVA revealed a main effect of the ethical information, *F* (2, 82) = 19.32, *p* < 0.001, *partial*η*^2^* = 0.32, supporting our third hypothesis—the ethical information was effective in shifting participants concern away from frugality and towards ethical concern. Pairwise comparisons showed the difference between the moderate anchor and the high anchor was significant (*p* < 0.001) confirming our fifth hypothesis—the high anchor was more effective than the moderate anchor. There was no main effect of target, *F* (1, 82) < 1, *p* = 0.533, *partial*η^2^ = 0.01.

**FIGURE 4:**
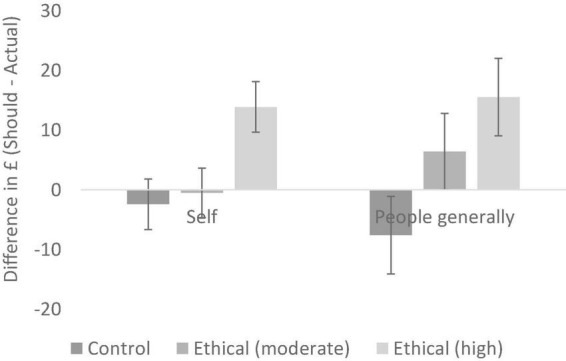
Should–actual differences for jeans (Experiment 2); error bars show 95% confidence intervals.

The main effect of ethical information was qualified by a significant interaction, *F* (2, 82) = 3.78, *p* = 0.027, *partial*η*^2^* = 0.08. To decompose this interaction, separate one-way ANOVAs were conducted for both levels of the target (see Figure 4). For the self, there was a main effect of condition, *F* (2, 82) = 17.67, *p* < 0.001, *partial*η*^2^* = 0.30. In this context, the moderate anchor (*M* = −0.52, *SD* = 10.27) did not shift participants away from the frugality default (*M* = −2.43, *SD* = 7.25, *p* = 1),^3^ but the high anchor did (*M* = 13.89, *SD* = 7.25, *p* < 0.001). For people generally, there was also a main effect of condition, *F* (2, 82) = 12.77, *p* < 0.001, *partial*η^2^ = 0.24. In this context, the moderate anchor (*M* = 6.41, *SD* = 15.81) significantly shifted participants away from the frugality default (*M* = −7.61, *SD* = 14.40, *p* = 0.009), with the high anchor eliciting this shift even more so (*M* = 15.54, *SD* = 20.94, *p* < 0.001).

Together, these data suggest that the high anchor mimicked the effect of the ethical information in Experiment 1, whilst the moderate anchor had a smaller effect. The moderate anchor was sufficient to shift should–actual differences for participants’ expectations of people generally (compared to the control group), yet a high anchor was required to shift the should–actual differences for the self. This pattern reflects participants’ different expectations for others compared to themselves; that is, the moderate anchor does not change the perception of *personal* gaps in should–actual behaviour, but it does change the perception of how *people generally* should act. Isolating the control and ethical (moderate) conditions and running the same analysis shows this interaction to be significant, providing further evidence for this assertion (see Supplementary Material for details). These data, therefore, support our fourth hypothesis.

##### Spillover variable (t-shirts)

The introduction of the price anchor for jeans in the ethical information in this experiment meant that t-shirts were now a spillover variable. Nonetheless, a mixed ANOVA still revealed a main effect of the ethical information, *F* (2, 82) = 10.13, *p* < 0.001, *partial*η^2^ = 0.20, on should–actual differences. Pairwise comparisons showed that the high anchor (*M* = 3.97 [1.33, 6.60]), elicited greater ethical concern, compared to the moderate anchor (*M* = −0.12 [−2.75, 2.51], *p* = 0.096) providing additional support for our fifth hypothesis. There was no main effect of target, *F* (1, 82) = 1.65, *p* = 0.202, *partial*η^2^ = 0.02.

The main effect of ethical information was qualified by a marginally significant interaction, *F* (2, 82) = 3.05, *p* = 0.053, *partial*η^2^ = 0.07. Separate one-way ANOVAs were again conducted for both levels of target. For the self, there was a main effect of condition, *F* (2, 82) = 4.36, *p* = 0.016, *partial*η^2^ = 0.10. The moderate anchor (*M* = −0.69, *SD* = 6.53) did not shift participants away from the frugality default (*M* = −3.81, *SD* = 5.72, *p* = 0.34), but the high anchor (*M* = 1.97, *SD* = 9.17) did (*p* = 0.012). For people generally, there was also a main effect of condition, *F* (2, 82) = 10.79, *p* < 0.001, *partial*η^2^ = 0.21. The moderate anchor (*M* = 0.45, *SD* = 7.24) marginally shifted participants away from the frugality default (*M* = −5.41, *SD* = 6.65, *p* = 0.057), and the high anchor even more so (*M* = 5.97, *SD* = 12.32, *p* < 0.001). These results replicate the findings for jeans: the moderate anchor attenuated the effect of the ethical information, whilst remaining somewhat more effective for people generally than for the self. Together, these data support our third, fourth, and fifth hypotheses.

Overall, we again found a default preference for frugality: participants expressed they, and others, spent more than they should. Although the pattern of data for self-other judgements was similar, the interpersonal hypocrisy effect from Experiment 1 was not fully replicated. That is, whilst the should–actual differences were larger for people generally compared to the self, the interaction was not significant. However, the data supported our third and fifth hypotheses, as we again found an inversion of default frugality and that the higher anchor was more effective than the moderate anchor at shifting participants in this direction. The data also supported our fourth hypothesis, as it was found that a moderate anchor was sufficient to shift participants’ expectations for what others should and actually do when it comes to purchasing clothes, but a high anchor was required to shift their own expectations.

## Experiment 3

In combination, the first two experiments provided promising evidence in support of our five hypotheses, but there was some inconsistency and we also wanted to test whether these effects would generalise outside United Kingdom student samples – which have their own particular lifestyle and financial concerns. Accordingly, the experiment was run in a larger public sample and a different country (United States). We also wanted to examine whether a more extreme anchor would produce potential ceiling or rebound effects found in other anchoring experiments (Mussweiler and Strack, 2001). Experiment 3, therefore, used four conditions (no anchor control, moderate anchor, high anchor, extra-high anchor) to test our five hypotheses. In relation to the fifth hypothesis, although we again expected the higher anchor to be more effective than the moderate anchor, we did not expect the extra-high anchor to produce equivalently stronger effects than the high anchor because it was intended to reach the boundary of plausibility (Mussweiler and Strack, 1999).

### Method

#### Participants

One-hundred and eighty-three participants based in the United States were recruited using Mechanical Turk (79 women, 102 men, 2 prefer not to say; *M*_age_ = 33.69, range 18–72 years). Each participant was paid $0.75. Incomplete entries were automatically rejected by the survey software. Exclusion criteria were used to eliminate participants who may have completed the survey multiple times or were not paying sufficient attention to the experiment. Seven participants (4%) used the same IP address, 13 (7%) failed an audio-visual video check, 6 (3%) failed a basic knowledge check, and 18 (10%) provided rare answers that suggested a lack of attention (e.g., other people should give *less* to charity than they actually do). In total, 38 participants were excluded, and because some (*n* = 6) failed on more than one of these basic checks, this left a final sample for analysis of 145.^4^

#### Ethical information

Participants in the ethical information conditions saw the same videos as presented in Experiment 2, with minor adjustments to reflect the American setting. Participants in the moderate anchor condition saw the anchor for the ethical production of jeans as $44, participants in the high anchor condition saw the anchor as $88, and participants in the extra-high anchor saw the anchor as $133.^5^ Participants in the control condition again simply proceeded to the dependent measures.

#### Design, procedure, and measures

A mixed design was used. The between-participants independent variable had four levels (no information, moderate anchor, high anchor, extra-high anchor). The within-participants dependent variable was again target (self vs. people generally). Participants received the same demographic items as before and were then presented with the relevant ethical information or no information. If they were presented with ethical information, a knowledge check item asked them to select the anchor they had seen from a list of options. Next, participants completed the should and actual contrasts for each item for themselves and then for people generally, followed by items assessing personal financial security, and their estimate of the cost of average jeans purchase for the typical American. The supplementary measures for charitable donations and moral judgement of cheap purchasing were again presented. The items assessing consumption were the same as in the previous experiments except for changes made to reflect US culture [e.g., using USD ($) and replacing tea with coffee]. For participants in the ethical information conditions, the knowledge check included an additional question asking them to select the type of music played in the video.

### Results and discussion

#### Frugality in the control condition

**FIGURE 5:**
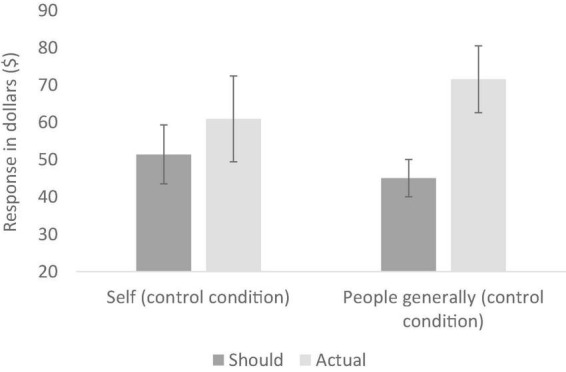
Reported should and actual spends for clothes in the two control conditions (Experiment 3); error bars show 95% confidence intervals.

In the control condition, we conducted a two-way repeated measures ANOVA with target (self vs. people generally) and type of judgement (should vs. actual spend on clothes) as the repeated measures variables. This analysis revealed a significant interaction, *F* (1, 40) = 8.28, *p* = 0.006, *partial*η^2^ = 0.17. As seen in Figure 5, participants thought they should spend (*M* = 51.41, *SD* = 25.04) a bit less than they actually do (*M* = 60.93, *SD* = 36.44), but this difference was not significant [*F* (1, 40) < 1, *p* = 0.615, *partial*η^2^ = 0.01]. In contrast, they thought people generally should spend (*M* = 45.05, *SD* = 15.88) a lot less than they actually do (*M* = 71.54 *SD* = 28.41), and this difference was significant [*F* (1, 40) = 58.61, *p* < 0.001, *partial*η*^2^* = 0.59]. These data confirm that the frugality gap participants expected for people generally was significantly larger than the gap they expected for themselves, supporting our first two hypotheses.

#### Effects of ethical information

We conducted a 4 (ethical information: control, moderate anchor, high anchor, extra-high anchor) × 2 (target: self vs. people generally) mixed ANOVA on should–actual differences for each item separately.

##### Primary variable (jeans)

The ANOVA revealed a main effect of ethical information, *F* (3, 134) = 15.96, *p* < 0.001, *partial*η*^2^* = 0.26 showing the default position of frugality had been inverted and providing direct support for our third hypothesis. However, pairwise comparisons showed the difference between the moderate anchor and the high anchor was not significant (*p* = 0.565), which did not directly support our fifth hypothesis.

**FIGURE 6:**
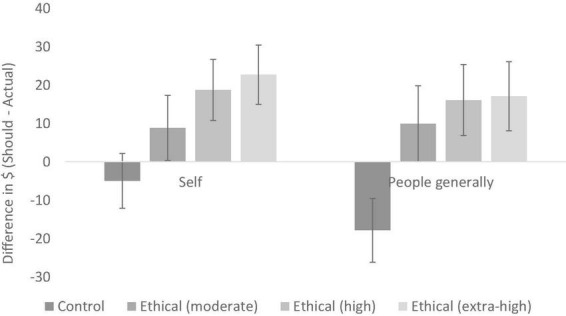
Should–actual differences for jeans (Experiment 3); error bars show 95% confidence intervals.

The main effect of ethical information was, however, qualified by a marginally significant interaction, *F* (3, 134) = 2.26, *p* = 0.085, *partial*η^2^ = 0.05 (see Figure 6). As with Experiment 2, the interaction was decomposed by conducting separate one-way ANOVAs for both levels of the target. For the self, there was a main effect of condition, *F* (3, 134) = 10.84, *p* < 0.001, *partial*η^2^ = 0.20. In this context, the moderate anchor (*M* = 8.79, *SD* = 13.37) only marginally shifted participants away from the frugality default (*M* = −5.00 *SD* = 17.36, *p* = 0.09), whilst the high anchor (*M* = 18.70, *SD* = 28.04) and extra-high anchor (*M* = 22.66, *SD* = 29.29) elicited a significant difference (both *p*s < 0.001). For people generally, there was also a main effect of condition, *F* (3, 134) = 14.44, *p* < 0.001, *partial*η^2^ = 0.24. The moderate anchor (*M* = 9.90, *SD* = 20.89) significantly shifted participants away from the frugality default (*M* = −17.90, *SD* = 19.09, *p* = 0.009), and the high anchor (*M* = 16.06, *SD* = 30.50, *p* < 0.001) and extra-high anchor even more so (*M* = 17.05, *SD* = 34.45, *p* < 0.001). These data replicate the finding from Experiment 2 that a moderate anchor has a stronger effect on participants’ expectations of others compared to themselves. Also replicating Experiment 2, isolating the control and ethical (moderate) conditions and running the same analysis show this interaction to be significant, providing further evidence for this assertion (see Supplementary Material for details). Together, these data provide support for our fourth hypothesis.

##### Spillover variable (t-shirts)

The ANOVA revealed a main effect of the ethical information, *F* (3, 138) = 12.30, *p* < 0.001, *partial*η^2^ = 0.21 and a main effect of the target, *F* (1, 134) = 6.88, *p* = 0.010, *partial*η^2^ = 0.05. However, there was no significant interaction, *F* (3, 138) = 1.83, *p* = 0.145, *partial*η^2^ = 0.04. This suggests the ethical information inverted the initial frugality default, but the specific level of anchor did not have a spillover effect. It is worth noting that pairwise comparisons showed the moderate anchor as only somewhat effective for shifting participants expectations of the self (*MD* = −7.55 [−15.38, 0.29], *p* = 0.066), but significantly effective for shifting participants’ expectations of others (*MD* = −12.09 [−19.92, −4.26], *p* < 0.001), in line with our fourth hypothesis.

Overall, the results from this public sample provided support for our first, second, third, and fourth hypotheses. Default frugality was again the norm and interpersonal hypocrisy was present in the control conditions. These effects were once more inverted by making ethical production issues salient. Whilst the anchors appeared equally effective if collapsed across conditions, the interaction showed that a high anchor was required to shift participants from their default position of personal frugality, whereas a moderate anchor was sufficient to shift participants’ expectations of what others should and actually do, just as in Experiment 2. Together, these effects suggest consistent findings of interpersonal hypocrisy. A ceiling effect of ethical demands may be present, given the high and extra-high anchors almost always carried the same effect. However, there was little evidence for a rebound effect, as the extra-high anchor did not significantly differ from the high anchor in any analysis.

## General discussion

The primary aim of this paper was to examine how two forms of hypocrisy could be evidenced in the context of ethical consumption. Across the three experiments, we found several findings in support of each of our five hypotheses. In relation to the first two hypotheses, with our novel methodology, we did not know beforehand if a frugality motive would be detected by our actual-ought price framework; and whilst for self-judgements, the effects were mixed and only reached statistical significance in Experiment 1, the overall finding is that, in the absence of other frames, people’s default expectation is we should be spending less on consumer items. Interpersonal hypocrisy was evident within this default position, as participants reported more profligacy among others than themselves.

In relation to the third hypothesis, this default frugality was reliably inverted after viewing relevant ethical information, such that the information made individuals believe they should spend more than they actually do. In relation to the fourth hypothesis, interpersonal hypocrisy was consistently evident within this inversion, as moderate anchors were sufficient to shift participants’ expectations of others’ spending, but higher anchors were required to shift participants’ expectations of themselves. And in relation to the fifth hypothesis, higher anchors produced a greater shift toward ethical spending than moderate anchors, although this difference was only significant in our United Kingdom sample.

The should–actual differences shown throughout our research reflect an acceptance of *intrapersonal* hypocrisy in purchasing contexts. However, we also demonstrate two important instances of *interpersonal* hypocrisy, one on each side of the aforementioned tension between cost and ethics. For cost, participants expected others to be less frugal than they themselves would be. For ethics, a higher anchor was required to shift participants toward a more ethical position for their own behaviour than was required for their expectations of others.

Interestingly, varying the target factor from between-participants (Experiment 1) to within-participants (Experiments 2 and 3) showed not only that participants have different expectations for themselves and others but also that they are willing to report these differences explicitly. This provides the first evidence we know of to illustrate a combination of intrapersonal and interpersonal hypocrisy effects in the use of ethical information and does so both when participants make these judgements independently and consecutively.

## Limitations and future research

Whilst our findings are provocative, we acknowledge that our research utilises a novel method for integrating intrapersonal and interpersonal hypocrisy and is therefore necessarily somewhat exploratory and contains several “moving parts.” Whilst across experiments, we have presented consistent evidence for how the frugality-ethics, should–actual, and self-other dimensions can interact, there were a couple of cases of inconsistency in support for our hypotheses within individual experiments. Future research would, therefore, be helpful to extend our understanding of how these processes interact in the contexts of ethical consumption, and beyond, particularly utilising higher-powered designs which offer greater potential for detecting some of the more subtle interactions that our experiments may have not been able to detect.

For example, we used anchoring in a within-participants design, but this may sometimes suppress people’s willingness to provide different standards for themselves and others. After people are provided with a specific and concrete numerical value within the ethical information, they have less “wriggle room” to justify any motivation to demand more of others. In contrast, individuals in the control conditions are not as constrained, although they may still be somewhat anchored by their initial judgements. This difference could explain why we find larger differences between targets in our control conditions, compared to our experimental conditions. Counterbalanced designs could be used in the future to specifically test whether consideration of one’s own or others’ behaviour has an impact upon the judgements participants make.

Future research could operationalise the tension between frugality and ethics in other ways, to further map out how differences between ought and actual behaviour vary between the self and others. For example, ought and/or actual behaviour could be measured independently. Furthermore, these methods could be used to gain a greater understanding of how intrapersonal and interpersonal hypocrisy work in conjunction with other variables that have been studied in the context of ethical consumption. Uncertainty (Milkman, 2012), product attributes (Luchs et al., 2010), knowledge (Mussweiler and Strack, 2000), thresholds for action (MacCoun, 2012), perceived agency (Antonetti and Maklan, 2014), emotional state (Polman and Ruttan, 2012; Ruedy et al., 2013), concern for sustainability (Balderjahn et al., 2013), group identity (Valdesolo and DeSteno, 2007), and ethical identity (Sparks and Shepherd, 1992; Oh and Yoon, 2014) are all constructs that have been shown to relate directly to ethical intentions. These associations could benefit from additional research that sets varying levels of thresholds for participants and examines how judgements of ought and actual behaviour vary as a function of the target (self vs. other).

A related point is the use of the extra-high anchor in Experiment 3. It was the only condition in either of the anchoring experiments where participants did not suggest a “should” value beyond the given anchor. This result suggests that the extra-high anchor was indeed at the boundary of plausibility. However, it was certainly not completely implausible, so it would be inappropriate to conclude what would happen if a completely implausible anchor was used in this experimental design. It may be that this would undermine the credibility of the ethical information and potentially the experiment itself. Such effects could produce the attenuation response that attitudinal research predicts for extreme anchors (Wegener et al., 2001), but perhaps more worryingly may prevent the participants from taking the research seriously. Future work would also benefit from testing price anchors in different contexts, to examine how the response scale being employed might impact upon people’s responses (Frederick and Mochon, 2012).

A similar method to ours could be employed in combination with other explanatory factors, to test for issues such as plausibility, persuasion, and conformity. For example, research could examine how ethical price anchors function in relation to individual differences. Advice tends to be used when it is already close to current perceptions (Yaniv, 2004). Consequently, baseline measures of existing beliefs and knowledge relating to ethical situations could help predict when different anchors are more effective. Personality variables that tap bases of product attitudes are also relevant. For example, those lower in self-monitoring are more persuaded by appeals that highlight the greater quality of the product (Snyder and DeBono, 1985) and, therefore, may be willing to spend more for such a product in response to high anchors. Although no effects of gender were found in our research, gender analyses are also of interest because of potential gender differences in ethical consumption (Shang and Peloza, 2015). It is also possible that individuals’ attitudes toward ethical consumption, in general, could be a useful additional predictor.

Finally, in our experiments, frugality was operationalised in terms of the motivation to spend less and contrasted against the motivation to spend more when ethical concerns were salient. However, this definition and our methods do not fully cover every aspect of frugality. In line with our above suggestions, further work could isolate the multiple aspects of the concept, such as consuming less (Lastovicka et al., 1999). Our novel approach shows new ways in which the tensions between price and ethics can be operationalised, but it does not inform all aspects of frugality, such as those relating to purchasing fewer goods. It would also be beneficial to address how cultural norms around frugality could moderate the tensions between price and ethics that we have illustrated, as some cultures are more likely to encourage consumer spending or inhibiting lavish purchasing than others (see Pan et al., 2019), and such differences would impact upon our operationalisations of frugality and hypocrisy.

## Conclusion

While some areas of ethical consumption have attracted considerable research, there is a clear need for further psychological explanations of how information about ethical consumption is perceived, elaborated upon, and acted upon. Our findings relating to frugality, anchoring, and hypocrisy offer a deeper understanding of the tensions people face when they try to navigate consumption in a globalised and complex marketplace. People have core motivations to be both frugal and ethical, and in the realm of consumption, these motives are often conflicting. Importantly, our research shows how people see others as needing to do more with regards to both of these motives, compared to themselves.

Given that price is often reported as the primary concern for those considering an ethical purchase (Bray et al., 2011) and that money is a psychologically powerful concept (Zhou et al., 2009), there is much merit in using price anchors and ethical consumption as an ecologically valid research model for further testing the tensions between concerns of the self versus others. Furthermore, higher pro-environmental consumption has been linked to higher life satisfaction, even controlling for demographic factors and environmental attitudes (Welsch and Kühling, 2010). If we can understand further how frugality, anchors, and hypocrisy work in the realm of ethical consumption, there are therefore potential benefits for producers, consumers, and the wider world.

## Data availability statement

The datasets presented in this study can be found in online repositories. The names of the repository/repositories and accession number(s) can be found in the article/Supplementary material.

## Ethics statement

The studies involving human participants were reviewed and approved by Cardiff University Ethics Committee. The patients/participants provided their written informed consent to participate in this study.

## Author contributions

CF and GM conceived the idea. CF created the materials, collected the data, and ran the data analyses. GM and GH supervised the work. All authors discussed the results and contributed to the final manuscript.

## Footnotes

1 The items for tea (and coffee in Experiment 3) were included to test for distant spillover effects, but for parsimony these analyses are reported in Supplementary Materials.

2 Exchange rate as at time of experiment.

3 The quoted p-values reflect Bonferroni’s adjustment and are thus capped at 1.

4 The sample size for each analysis will vary as participants were asked not to respond if any item was not relevant to them, e.g., if they did not buy jeans or t-shirts.

5 US prices for premium brand jeans were around $80 at the time of data collection.
