# Supplementary material for: Hypocrisy in ethical consumption
Source: Front Psychol. 2022 Aug 25;13:880009. doi: 10.3389/fpsyg.2022.880009 (PMC9453667; doi:10.3389/fpsyg.2022.880009)
Supplement: Supplementary file 1 [file Data_Sheet_1.docx]

**Supplementary Materials**

**EXPERIMENT 1**

**Distant spillover variable (tea)**.

The ANOVA revealed a significant main effect of ethical information, *F* (1, 68) = 18.89, *p* < .001, *partial η^2^* = .22 but the main effect of target (*p* = .496) and the interaction were non-significant (*p* = .812). The ethical information elicited significant changes for both the self (*MD* = 1.15, *p* = .004, *partial η^2^* = .12) and for people generally (*MD* = 1.29, *p* = .002, *partial η^2^* = .13). These data show the impact of the ethical information did spillover into a product not mentioned in the intervention, but the shift from expectations of frugality to ethics was equal for both targets.

**Charitable Donation**

As shown in Figure S1, unlike the default position for consumer items (frugality), the reverse position was adopted for donating to charity. Perhaps unsurprisingly, 95% of participants in the control condition suggested they should donate more to charity on a monthly basis. The ANOVA revealed a significant main effect of the ethical information, *F* (1, 77) = 7.35, *p* = .008, *partial η2* = .09. Participants who saw the ethical information suggested the gap between how much they, or people generally, should and actually donate to charity was larger compared to those who did not see the information. The main effect of target was non-significant, (*p* = .438). However, the interaction was significant, *F* (1, 77) = 3.96, *p* = .050, *partial η2* = .05. As seen in Figure S1, simple effects analyses revealed that the effect of the ethical information was significant for those asked about what *people generally* should and actually donate to charity, *F* (1, 77) = 10.65 *p* = .002, *partial η2* = .12, whereas it was non-significant for those asked about their *own* charity donations, *F* (1, 77) < 1, *p* = .605, *partial η2* < .01. These data suggest the video changed participants’ views on whether other people should increase their charitable donations, but it did not change their views on their own donation behaviour.

*Figure S1: Should-actual differences for monthly charity donations (Experiment 1); error bars show 95% confidence intervals*

**Moral Judgement**

An ANOVA on participants’ judgements of the morality of a student who paid £8.50 for a pair of jeans revealed a significant main effect of ethical information, *F* (1, 79) = 13.44, *p* < .001, *partial η^2^* = .15. Participants who saw the information were more likely to see the purchasing of cheap jeans as morally wrong (*M* = 8.31; [7.61, 9.01]), than the control group (*M* = 6.50; [5.81, 7.19]). The main effect of target and the interaction were non-significant (both *p*s > .293). Thus, the ethical information led to increased moral concern about a cheap purchase.

**Estimate of Average Spend on Jeans**

An ANOVA on the average student spend on jeans revealed a significant main effect of the ethical information, *F* (1, 79) = 4.16, *p* = .045, *partial η^2^* = .05. Participants who saw the information estimated a lower average spend on jeans (*M* = £21.91; [18.85, 24.96]), compared to those who did not see the information (*M* = £26.36; [23.27, 29.45]). The main effect of the target and the interaction were not significant (both *p*s > .434). Across conditions, participants perceived the average jeans price paid by a student to be approximately £24, which we used in the next experiment for setting appropriate anchors.

**EXPERIMENT 2**

**Primary variable (jeans); additional analysis**

To further test whether the moderate anchor was shifting participants’ expectations from default frugality for people generally, but not for themselves, we ran an additional analysis which isolated the control and ethical (moderate) conditions. The interaction between target and condition was significant, *F* (1, 55) = 7.45, *p* = .009 *partial η^2^* = .12, providing additional evidence for this hypothesis.

**Distant spillover variable (tea)**.

The ANOVA revealed a main effect of the ethical information, *F* (2, 54) = 5.75, *p* = .015, *partial η^2^* = .18, but no significant interaction between information condition and target (*p =* .948). The significant main effect suggests the information still had a spillover effect outside of its specific context. Participants in the high anchor group had the largest should-actual contrast (*M* = 1.08 [95 CIs: 0.58, 1.58]), followed by the moderate anchor group (*M* = 0.35 [-0.10, 0.80]). The control group again had a negative mean (*M* = -0.16 [-0.73, 0.39]), which indicates a preference for frugality, though the confidence intervals contain zero reflecting a potential underlying default motivation for ethical consumption particular to this product. The lack of interaction here thus seems driven by the lack of a frugality default in the control group for people generally, hence participants were not automatically construing the same tension between frugality and ethics that they were for clothes. This is the only instance where participants in the control group suggested people should spend *more* than they actually do, probably reflecting extant norms for this particular product (it is far more common to see ethical branding for tea than clothes). Importantly, this further shows the importance of using a ‘no information’ control group as a comparison for each product, as some items may be more likely to provoke automatic motivations, such as frugality or ethical concern, than others.

**Charitable Donation**

No significant effects were found for charitable donations (all *p*s > .118). However, the data trended similarly to the effects found in Experiment 1 - the high threshold video showed the greatest gaps between actual and should behaviour, particularly for people generally.

*Figure S2: Should-actual differences for monthly charity donations (Experiment 2); error bars show 95% confidence intervals*

**Moral Judgement**

A one-way ANOVA revealed a main effect of video condition on the moral judgement of buying cheap jeans, *F* (2, 84) = 8.98, *p <* .001, *partial η^2^* < .18. Pairwise comparisons revealed that participants thought the act was significantly more morally wrong after seeing the moderate threshold video (*M* = 5.73 [4.96, 6.51]) or the high threshold video (*M* = 5.93 [5.15, 6.71]), compared to the control group (*M* = 7.89 [7.09, 8.69]). Similar results were found for the cheap tea purchase, *F* (2, 83) = 7.64, *p* = .001, *partial η^2^* < .16. The spillover effects found for ethical consumption items in Experiment 1 thus appear to extend to moral judgements of others.

**Estimate of Average Jeans Spend**

As with Experiment 1, the final item asked participants to estimate the price students would normally pay for an average pair of jeans. A one-way ANOVA revealed a marginally significant main effect of ethical information condition on this estimate, *F* (2, 84) = 2.73, *p* = .071, *partial η^2^* = .06. Pairwise comparisons revealed participants thought students spent marginally less (*p* = .087) having seen the moderate anchor (*M* = £21.13 [17.77, 24.50]), compared to the control group (*M* = £26.54 [23.06, 30.02]). The difference between the moderate anchor and the high anchor (*M* = £25.28 [21.86, 28.70]) was not significant (*p* = 0.269). This is similar to the effect in Experiment 1.

**EXPERIMENT 3**

**Primary variable (jeans); additional analysis**

To further test whether the moderate anchor was shifting participants’ expectations from default frugality for people generally, but not for themselves, we ran an additional analysis which isolated the control and ethical (moderate) conditions. The interaction between target and condition was significant, *F* (1, 68) = 5.42, *p* = .023 *partial η^2^* = .07, providing additional evidence for this hypothesis.

**Distant spillover variable (coffee)**.

The ANOVA revealed a main effect of ethical information, *F* (3, 93) = 7.01, *p* = .009, *partial η^2^* = .07. Pairwise comparisons showed the frugality default position in the control condition (*M* = -1.80, [-3.31, -0.29]) was changed by the moderate anchor (*M* = 1.98, [0.28, 3.67], *p* = .008), the high anchor (*M* = 2.51, [0.82, 4.20], *p* =.002), and the extra-high anchor ((*M* = 1.74, [0.01, 3.47], *p* = 0.17). However, all the anchors were equally effective across target, as no differences were found between ethical information conditions (all *p*s =1). So, whilst we saw the predicted inversion as a result of the ethical information, we did not see different effects across the anchoring conditions.

The main effect of ethical information was qualified by a marginally significant interaction, *F* (3, 93) = 4.26, *p* = .007, *partial η^2^* = .12. As with Experiment 2, to decompose this interaction, separate one-way ANOVAs were run for both levels of target. For the self, there was a main effect of condition, *F* (3, 93) = 4.00, *p* = .010, *partial η^2^* = .11. The moderate anchor (*M* = 1.35, *SD* = 4.21) and the high anchor (*M* = 1.39, *SD* = 5.70) did not shift participants significantly (*p*s > .146) from their frugality default (*M* = -1.38 *SD* = 2.56), but the extra-high anchor (*M* = 2.61, *SD* = 4.67) did make a significant difference (*p* = 009). For people generally, there was also a main effect of condition, *F* (3, 93) = 7.24, *p* < .001, *partial η^2^* = .19. The moderate anchor (*M* = 2.61, *SD* = 4.67, *p* = .004) and the high anchor (*M* = 3.63, *SD* = 6.59, *p*  < .001) significantly shifted participants from the frugality default (*M* = -2.22, *SD* = 3.20), but the extra-high anchor did not (*M* = 0.86, *SD* = 3.81, *p* = .168). These data replicate the finding from Experiment 2, that a moderate anchor has a stronger effect for participants’ expectations of others compared to themselves.

**Charitable Donation**

The ANOVA revealed no main effect of the ethical information video, *F* (3, 136) < 1, *p* = .507, *partial η^2^* = .02 but a main effect of the hypocrisy target, *F* (1, 136) = 10.48, *p* = .002, *partial η^2^* = .07. Across conditions, participants thought people generally should increase their charity donations more than they themselves should – another interpersonal hypocrisy effect. However, there was no significant interaction, *F* (3, 136) = 1.75, *p =* .159, *partial η^2^* = .04.

*Figure S3: Should-actual differences for monthly charity donations (Experiment 3); error bars show 95% confidence intervals*

**Moral Judgement**

A one-way ANOVA revealed a main effect of video condition on the moral judgement of buying cheap jeans, *F* (3, 141) = 9.51, *p <* .001, *partial η^2^* = .17. Further analysis revealed participants thought the act was less morally acceptable after seeing the moderate threshold video (*M* = 5.69, [4.87, 6.50], *p* < .001), the high threshold video (*M* = 6.65; [5.86 7.44], *p* = .006) and the extra-high threshold (*M* = 6.54, [5.78, 7.30], *p* = .002), compared to the control group (*M* = 8.45, [7.74, 9.16]). Similar results were found for the cheap coffee purchase, *F* (3, 141) = 6.47 *p <* .001, *partial η^2^* = .12. Participants again thought the act was significantly more morally questionable after seeing the moderate threshold video (*M* = 5.81, [4.99, 6.64], *p* <.001), the high threshold video (*M* = 6.68, [5.87, 7.48], *p* = .043) and the extra-high threshold (*M* = 6.68, [5.91, 7.45] *p* = .036), compared to the control group (*M* = 8.17, [7.44, 8.89]). Similar to the previous two studies, the control group participants rated the behaviour as morally acceptable, whilst those who saw any of the ethical information videos moved towards the midpoint (*neutral*) of the scale.

**Estimate of Average Jeans Spend**

A one-way ANOVA revealed no effect of ethical information condition on the estimated purchase price of an average pair of jeans, *F* (3, 141) < 1, *p =* .625, *partial η^2^* = .01. Unlike the previous experiments, there was no indication that the manipulation had changed the perceived average spending on jeans.

**VIDEO MATERIAL LINKS**

Experiment 1: https://www.youtube.com/watch?v=-nbIGHKip6o

Experiment 2 (moderate): https://www.youtube.com/watch?v=PbuKPCILBXQ

Experiment 2 (high): https://www.youtube.com/watch?v=TPoDrNSln84

Experiment 3 (moderate): https://www.youtube.com/watch?v=LNFU9KypqZc

Experiment 3 (high): https://www.youtube.com/watch?v=x0OhYa3c3UY

Experiment 3 (extra-high): https://www.youtube.com/watch?v=5gnVROLjmXE

**OPEN SCIENCE**

The data for each experiment are available on the Open Science Framework at this address:

https://osf.io/e6g5d/?view_only=b5548e7c92f54227a9f8a72ca379c89c
